# ENO1 contributes to 5-fluorouracil resistance in colorectal cancer cells *via* EMT pathway

**DOI:** 10.3389/fonc.2022.1013035

**Published:** 2022-12-22

**Authors:** Jinrong Gu, Kaiqiang Zhong, Longgang Wang, Haishun Ni, Yirui Zhao, Xuchao Wang, Yizhou Yao, Linhua Jiang, Bin Wang, Xinguo Zhu

**Affiliations:** ^1^ Department of General Surgery, the First Affiliated Hospital of Soochow University, Suzhou, Jiangsu, China; ^2^ Department of Emergency Medicine, the First Affiliated Hospital of Soochow University, Suzhou, Jiangsu, China

**Keywords:** ENO1, 5-FU, drug resistance, EMT pathway, colorectal cancer

## Abstract

**Introduction:**

Chemoresistance is a major barrier in the treatment of colorectal cancer (CRC) and many other cancers. ENO1 has been associated with various biological characteristics of CRC. This study aimed to investigate the function of ENO1 in regulating 5-Fluorouracil (5-FU) resistance in CRC.

**Methods:**

ENO1 level in 120 pairs of tumor tissues and adjacent normal tissues was examined by immunohistochemistry, and the correlation between ENO1 expression and prognosis was explored by survival analysis. Its role and potential mechanisms in regulating 5-FU resistance in CRC were studied by Western blotting, MTT assay, colony formation assay and transwell invasion assay. Murine xenograft assay was implied to verify the results *in vivo*.

**Results:**

Our study indicated that ENO1 was elevated in CRC tissues and was associated with poor patient prognosis. High levels of ENO1 expression were detected as a significant influencing factor for overall survival. Furthermore, ENO1 expression was found to have increased in drug-resistant cells (HCT116/5-FU and SW620/5-FU) constructed by increasing concentrations of 5-FU. Knockdown of ENO1 markedly increased the drug susceptibility and inhibited the proliferation and migration ability of HCT116/5-FU and SW620/5-FU cells. It was found that down-regulation of ENO1 inhibited the epithelial-mesenchymal transformation (EMT) signaling process. Finally, a murine xenograft assay verified that the depletion of ENO1 alleviated 5-FU resistance.

**Conclusion:**

This study identified that ENO1 regulated 5-FU resistance via the EMT pathway and may be a novel target in the prevention and treatment of 5-FUresistant CRC.

## Introduction

Colorectal cancer (CRC) is the world’s fourth most deadly cancer with almost 900 000 deaths reported annually in recent years (1). Accordingly, additional examinations and treatment strategies are being applied to cope with this global health burden. Application of new drugs has nearly doubled the average survival time for patients with advanced CRC over the past few years. However, for most patients, the overall survival duration remains to be less than 3 years (2). Drug resistance is a crucial factor with regard to this phenomenon. Mechanisms of drug resistance include tumor burden and growth kinetics, physical barriers, the immune system, and tumor microenvironment (3). However, the specific mechanism of chemoresistance with respect to 5-fluorouracil (5-FU) in CRC needs further investigation.

Enolase 1 (ENO 1) is a type of enolase isozyme that promotes the formation of phosphoenolpyruvate *via* catalysis by 2-phospho-glycerate and generates ATP during glycolysis (4). It is a glycolytic enzyme with multiple functions in bacterial and fungal infections; autoantigen activities; and occurrence, development and metastasis of cancer (5, 6). ENO1 was also reported to be a plasminogen receptor involved in the invasion and metastasis of pancreatic ductal adenocarcinoma (PDA) cells (7, 8). ENO1 overexpression has been demonstrated in a wide range of human cancers and is often associated with poor prognosis. In some cancer patients, increased expression of ENO1 is often accompanied by the production of anti-ENO1 autoantibodies, making the protein a tumor-associated antigen (9). It has been discovered that ENO1 expression levels were associated with tumorigenesis, proliferation, metastasis, and stem cell-like properties in various types of cancer (10, 11).

ENO1 is considered to be a potential target in novel immunotherapies and inhibiting it could contribute to the eradication of tumors to some extent (12). A previous study indicated that overexpression of ENO1 could enhance the resistance toward tamoxifen, whereas decreased expression of ENO1 could exhibit the opposite result in human breast cancer cells (13). Further, ENO1 knockdown can sensitize hypoxia-induced chemical resistance in pancreatic cancer cells by regulating REDOX homeostasis, which may be associated with an increase in intracellular reactive oxygen species levels, affecting cell cycle and proliferation (14). However, the relationship between ENO1 and resistance toward 5-FU in CRC is still unclear.

Epithelial-mesenchymal transformation (EMT) is a biological process wherein epithelial cells are transformed into cells with mesenchymal phenotypes through a specific procedure. EMT is mainly induced by EMT-activated transcription factors (EMT-TFs) from the SNAIL, TWIST, and ZEB families, resulting in the loss of cell polarity (15, 16). Such structural changes play an important role in embryonic development, e.g., by promoting the formation of the stomach, the layering of the neural crest, and the generation of different types of cells and tissues. However, abnormal activation of EMT stimulates the progression of pathological processes such as cancer development and fibrosis (17). EMT acts as a key factor in various biological processes, especially in tumorigenesis, proliferation, metastasis, and drug resistance during cancer development (18, 19). In addition, EMT is closely associated with chemotherapeutic drug resistance, e.g., 5-FU resistance in CRC, sorafenib resistance in hepatocellular carcinoma, and anti-EGFR therapy resistance in non–small cell lung cancer (NSCLC) (20–23).

In brief, our study aimed to demonstrate that ENO1 can modulate chemoresistance toward 5-FU in CRC *via* the EMT pathway. Thus, ENO1 may have application value in alleviating drug resistance in CRC.

## Methods

### Human tissue samples

In all, 120 pairs of tumor and adjacent tissues were obtained from postoperative specimens of patients from the General Surgery Department of the First Affiliated Hospital of Soochow University (Suzhou, China) between 2015 and 2016. The patient did not receive neoadjuvant chemoradiotherapy before surgery and all specimens were pathologically confirmed. The study was approved by the Independent Ethics Committee of the First Affiliated Hospital of Soochow University (2020076), and written informed consent was acquired from all patients.

### Cell culture

Human colon carcinoma HCT116 and SW620 cells were purchased from Cell Bank of the Chinese Academic of Sciences (Shanghai, China) and HT29, SW480, and LOVO cells were collected from Central Laboratory of the First Affiliated Hospital of Soochow University. We obtained 5-FU-resistant HCT116 (HCT116/5-FU) and 5-FU-resistant SW620 (SW620/5-FU) cells by treating HCT116 and SW620 cells with gradually increasing concentrations of 5-FU for 6 months. All cells were cultured in RPMI 1640 medium (Hyclone) containing 10% fetal bovine serum (Gibco, USA), penicillin G sodium (100U/ml) and streptomycin (100μg/ml). Cells were preserved at 37°C with 5% CO_2_. HCT116/5-FU and SW620/5-FU cells were transfected with ENO1-shRNA according to our previous study (24). Transient silencing was confirmed by Western blotting. HCT116/5-FU and SW620/5-FU cells were also treated with 2-deoxyglucose (MedChemExpress) at 20 mM for 4 h before analysis (25).

### Immunohistochemical analysis

Immunohistochemical staining was performed on tumor tissue slides as previously described (24). The sections were incubated with 1:100 diluted polyclonal anti-human ENO1 (BOSTER, Wuhan, China) at 4°C overnight. The staining intensity was scored from 0 to 4 (<5% = 0, 5% to <25% = 1, 25% to <50% = 2, 50% to <75% = 3, ≥ 75% = 4) during semi-quantitative evaluation. The staining score of each sample was decided by two authors in a blinded manner.

### Western blotting

Protein samples were obtained with the help of lysis buffer supplemented with protease and phosphatase inhibitors. Sodium dodecyl sulfate polyacrylamide gel electrophoresis was applied for protein separation. Subsequently, the samples were transferred onto polyvinylidene fluoride (PVDF) membranes. After blocking with 5% non-fat milk for 1 h, the PVDF membrane was incubated with antibodies at 4°C with gentle shaking, followed by treatment with horseradish peroxidase-conjugated secondary antibodies. Anti-ENO1 (Boster, Wuhan, China) and anti-β-Actin (1:5000, Bioss, Beijing, China) were the primary antibodies. We used Image J software to assess the picture quality of Western blotting.

### Cell viability assay

Cell viability was detected by Methyl Thiazolyl Tetrazolium (MTT) assay according to the manufacturer’s instructions. In brief, we seeded 2000 transfected cells in 96-well plates, and cells in each plate were cultured for different durations (12, 24, 36, 48, 60, and 72 h). The MTT solution was added to each plate for 4 h, and 150 μL of dimethyl sulfoxide was then added to each well with 10 min of gentle shaking. Subsequently, the absorbance in each well was measured at 490 nm (A490) for sample detection.

### Colony formation assay

Here 1000 cells with ENO1 expression knockdown or vectors were seeded in 6-well plates and cultured in a drug-free medium for 10 days until visible colonies formed. The formed clones were fixed with methanol, stained with crystal violet solution, and counted *via* microscopy.

### Transwell invasion assay

Cells were added to the top chambers at the density of 10,000 cells/200 µl, while the bottom chambers were filled with culture medium in each well. After incubation for 12 h at 37°C, the inserts were fixed and stained with crystal violet and the number of invading cells was counted randomly under a microscope.

### Murine xenograft assay

SPF male mice (4 weeks old, weighing approximately 18 g) were acquired from Shanghai SLRC laboratory Animal Co. Ltd. (Shanghai, China). All animal experiments were approved by the First Affiliated Hospital of Suzhou University Ethics Committee. In all, 24 mice were randomly divided into four treatment groups: ENO1-Kockdown (KD), Negative control (NC), ENO1-Knockdown (KD) with 5-FU, and Negative control (NC) with 5-FU. Subsequently, the mice were subcutaneously injected with ENO1-KD or NC-shRNA HCT116, depending on the group, into the left neck back on day 0. A total of 50 mg/kg of 5-FU was intraperitoneally injected into the 5-FU-treated mice every 3 days. The other two groups received saline treatment. The subcutaneous tumor size was measured every three days and mice were killed on the 21^st^ day. The tumors were then photographed.

### Statistical analysis

All experiments were repeated at least three times, and the results are presented as mean ± standard deviation (SD). Student’s T-test (unpaired, two-tailed) was used to compare means between two groups. IHC analysis results were evaluated by Chi-squared test or Fisher’s exact test. SPSS 25.0 and GraphPad Prism 8.0 were applied to analyze most of the data. Survival curves were analyzed using Hiplot (https://hiplot-academic.com/) to investigate the prognostic value of ENO1. Self-developed R program (version 3.6.1 for Windows, http://cran.r-project.org/) was used for Cluster analysis and Nomogram analysis. P < 0.05 was considered statistically significant.

## Results

### ENO1 is upregulated in CRC tissues and results in poor patient prognosis

**Figure 1:**
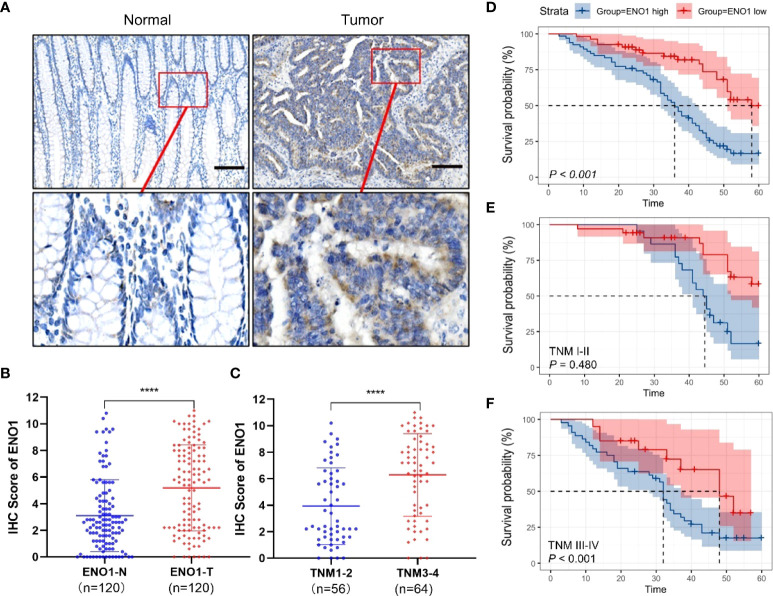
Expression of ENO1 in CRC tissues. **(A)** Representative immunohistochemical (IHC) images showing *in situ* ENO1 expression in colorectal cancer (CRC) and normal tissues (scale bar = 100 μm). **(B)** IHC scores of ENO1 in CRC vs normal tissues. **(C)** IHC scores of ENO1 in T I-II vs T III-IV tissues. **(D–F)** Overall survival of CRC patients with high and low expressions **(D)** of ENO1 with TNM staging I-II **(E)** and III-IV **(F)**. Statistical analyses were performed via two-tailed Student t test. ****, P ≤ 0.001.

The ENO1 protein expression was significantly increased in CRC tissues compared with that in normal colorectal tissues (**Figures 1A, B**). Tissues with tumor-node-metastasis (TNM) staging I-II showed lower ENO1 expression compared with those with TNM staging III-IV in CRC (P < 0.01; **Figure 1C**). Thus, ENO1 expression was found to be significantly associated with lymph node invasion (P =0.001), nerve invasion (P<0.05), vascular invasion (P<0.05), and TNM staging (P = 0.001, **Table 1**), whereas variables such as age, gender, tumor size, tumor location, or degree of differentiation were not considered to have much association with ENO1 expression (P > 0.05; **Table 1**). Univariate analysis demonstrated that the depth of invasion (P<0.01), lymph node metastases (P<0.05), nerve invasion (P<0.05), TNM stage (P<0.05, **Table 2**), and ENO1 expression were associated with worse survival. Moreover, multivariate analysis showed that high ENO1 expression was detected as a significant influencing factor for patient survival in CRC (P < 0.01, **Table 2**). Patients were divided into two groups according to ENO1 expression levels, and we found that patients in the high ENO1 group had a shorter lifespan than those in the low ENO1 group (P < 0.001; **Figure 1D**). Similarly, elevated ENO1 expression levels were associated with poor prognosis in patients with TNM staging III-IV but not those with staging I-II (P <0.001; P = 0.48; **Figures 1E, F**).

**Table 1:** Relationship between ENO1 and clinic-pathological factors in CRC patients.

Clinic parameters	Case	ENO1 expression		p value
		low	high	
Age				
<65	51	26	25	0.258
≥65	69	28	41	
Gender				
Male	59	28	31	0.595
Female	61	26	35	
Tumor size				
<5cm	55	28	27	0.231
≥5cm	65	26	39	
Tumor location				
Right	27	11	16	0.816
Left	30	13	17	
Rectum	63	30	33	
Differentiation				
Well	83	37	47	0.592
Poor	37	18	19	
Depth of invasion				
T1-2	42	22	20	0.233
T3-4	78	32	46	
Lymph node metastasis				
N0	56	34	22	0.001
N1-2	64	20	44	
Nerve invasion				
Yes	77	40	37	0.041
No	43	14	29	
Vascular invasion				
Yes	72	38	34	0.036
No	48	16	32	
TNM stage				
I/II	56	34	22	0.001
III/IV	64	20	44	

P-values < 0.05 were considered statistically significant.

**Table 2:** Results of univariate and multivariate analyses of postoperative patients’ survival by Cox’s proportional hazard model.

Varieties	n	Univariate analysis			Multivariate analysis		
		P	HR	95% CI	P	HR	95% CI
Age (<65/≥65)	51/69	0.166	0.712	0.441-1.151			
Gender (Male/Female)	59/61	0.519	0.857	0.537-1.369			
Tumor size(<5cm/≥5cm)	55/65	0.971	0.991	0.621-1.582			
Differentiation (Well/Poorly)	83/37	0.955	1.014	0.613-1.679			
Depth of invasion (T1-2/T3-4)	42/78	0.005	0.469	0.276-0.795	0.006	0.472	0.277-0.806
Lymph node metastasis (N0/N1-2)	56/64	<0.001	0.407	0.250-0.661	0.013	0.529	0.321-0.872
Nerve invasion (Yes/No)	77/43	0.012	0.543	0.337-0.875	0.037	0.598	0.369-0.970
Vascular invasion (Yes/No)	72/48	0.167	0.719	0.450-1.148			
TNM stage (I-II/III-IV)	56/64	<0.001	0.407	0.250-0.661	0.013	0.529	0.321-0.872
ENO1 (low/high)	54/66	<0.001	0.316	0.187-0.535	<0.001	0.602	0.459-0.791

HR, hazard ratio; CI, confidence interval.

**Figure 2:**
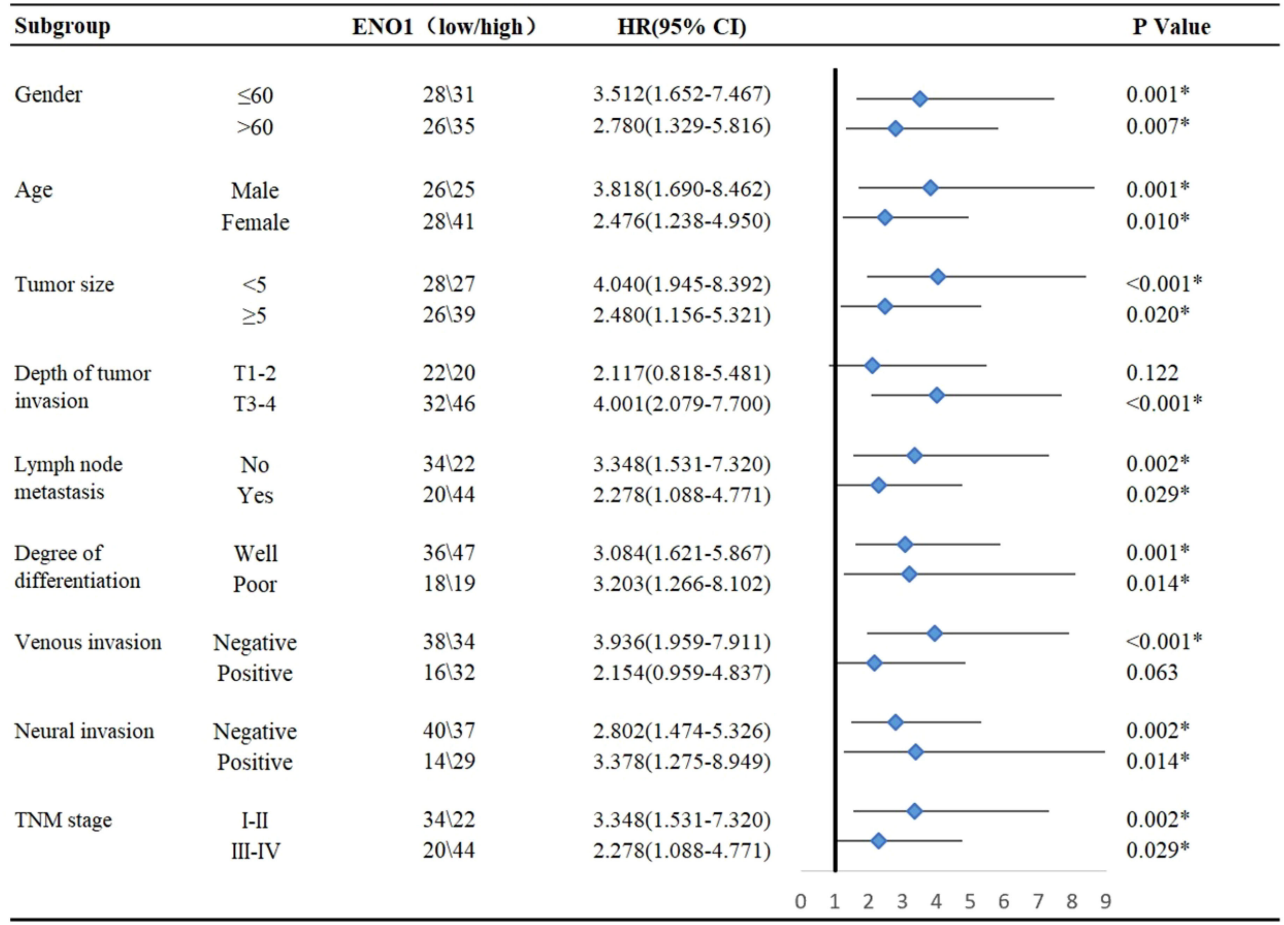
Subgroup analysis for the influencing factor of survival among colorectal cancer patients according to ENO1 expression. Statistical analyses were performed via two-tailed Student t test. *, P ≤ 0.05.

Subgroup analysis showed that high ENO1 expression was associated with worse prognosis, regardless of age, gender, lymph node metastasis, degree of differentiation, neural invasion, and TNM stage (P<0.05, **Figure 2**). In contrast, ENO1 expression level had no correlation with depth of tumor invasion T1-2 (P = 0.122) and venous invasion positivity (P = 0.063, **Figure 2**). Therefore, ENO1 expression has certain significance in analyzing and conjecturing patients survival with different depths of tumor invasion and degree of venous metastasis.

### ENO1 was up-regulated in fluorouracil-resistant CRC cells

**Figure 3:**
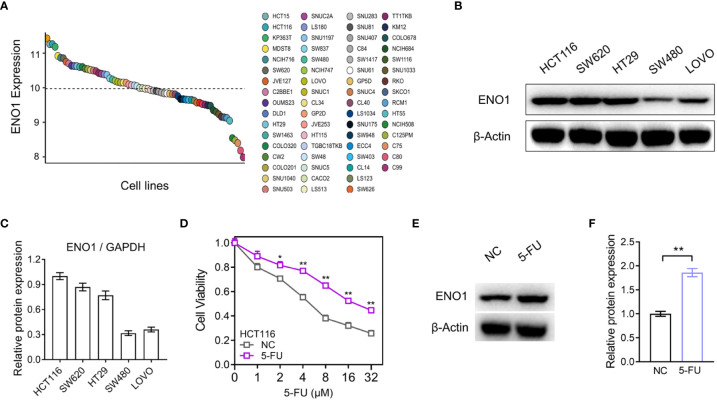
ENO1 expression was increased in the constructed 5-fluorouracil (FU)-resistant cells. **(A)** Comparison of ENO1 expression between various CRC cell lines in Cancer Cell Line Encyclopedia (CCLE). **(B, C)** Immunoblotting results of ENO1 **(B)** in cell lines HCT116, SW620, HT29, SW480, and LOVO and quantitative counting **(C)**. **(D)** Cell viability comparison between HCT116 cells and 5-FU-resistant cells. **(E, F)** Increase in ENO1 expression **(E)** in 5-FU-resistant cells and the statistical graph **(F)**. Statistical analyses were performed *via* two-tailed Student t test. *,P ≤ 0.05; **,P ≤ 0.01.

The expression of ENO1 in dozens of intestinal cancer cell lines was evaluated in Depmap Portal, online database of Cancer Cell Line Encyclopedia (CCLE) (**Figure 3A**). Subsequently, we performed immunoblotting in HCT116, SW620, HT29, SW480, and LOVO cell lines and the result showed that ENO1 expression in HCT116 and SW620 cell lines was higher than that in the other three cell lines, consistent with the trend shown in **Figure 3A**. Therefore, these cell lines were selected for further study (**Figures 3B, C**). We observed that cell viability of 5-FU-resistant cells was higher than that of negative control cells at the same concentration, indicating that the resistant group cells were more tolerant to 5-FU (**Figure 3D**). A similar trend was also observed in SW620-116/5-Fu. Compared with the NC cells, the expression of ENO1 in 5-FU-resistant cells was significantly increased (**Figures 3E, F**). These results demonstrated that ENO1 may be involved in 5-FU drug resistance and may act as a predictor for chemotherapy outcomes in CRC.

### Down-regulation of ENO1 inhibited the proliferation of 5-FU-resistant CRC cells *in vitro*

**Figure 4:**
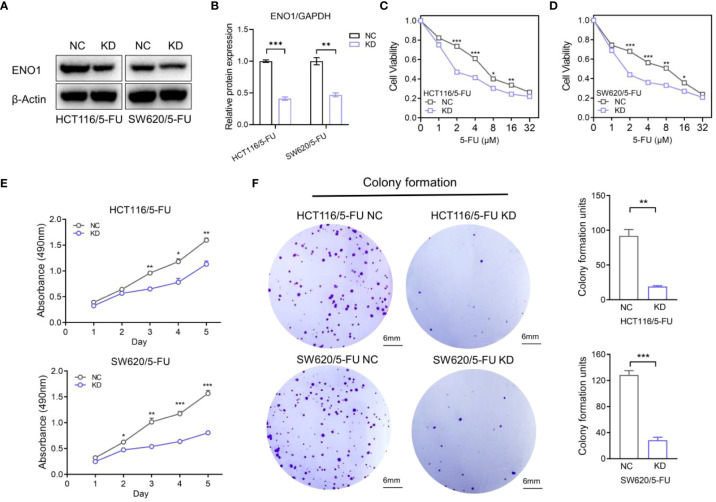
Knockdown of ENO1 inhibited the proliferation of 5-fluorouracil (FU)-resistant colorectal cancer (CRC) cells. **(A, B)** Immunoblots **(A)** showing ENO1 protein levels in NC and ENO1-KD group and the quantified pattern **(B)**, reflecting the success of transfection. **(C, D)** Cell viability of HCT/5-FU **(C)** and SW620/5-FU **(D)** cells in the ENO1-KD group and NC group. **(E, F)** Proliferation rates **(E)** and colony forming capacity **(F)** of 5-FU-resistant CRC cells in the ENO1-KD and NC groups (scale bar = 6mm). Statistical analyses were performed *via* two-tailed Student t test. *,P ≤ 0.05; **,P ≤ 0.01; ***,P ≤ 0.001.

HCT-116/5-FU and SW620/5-FU cells were transfected with ENO1-shRNA or NC-shRNA, following which ENO1 expression was significantly decreased compared with that in NC cells, and the most representative pattern is shown (**Figure 4A, B**). Subsequently, we explored whether the silencing of ENO1 influenced 5-FU tolerance in drug-resistant cells. The cell viability in the ENO1-KD group was significantly decreased compared with that in the NC group, indicating that the ENO1-KD group was more sensitive toward 5-FU. The results suggested that the down-regulation of ENO1 reversed fluorouracil resistance in CRC (**Figures 4C, D**). Consistent with this, MTT assay showed that the absorbance in the KD group was lower than that in the NC group, which illustrated that the proliferative ability of drug-resistant cells was significantly decreased (**Figure 4E**). Similarly, the colony-formation ability was suppressed in HCT-116/5-FU and SW620/5-FU cells following ENO1 knockdown compared with that in the negative control group (**Figure 4F**). The trend was shown more obviously in quantitative display.

### ENO1 promoted migration in 5-FU-resistant CRC cells and is possibly closely associated with the EMT pathway

**Figure 5:**
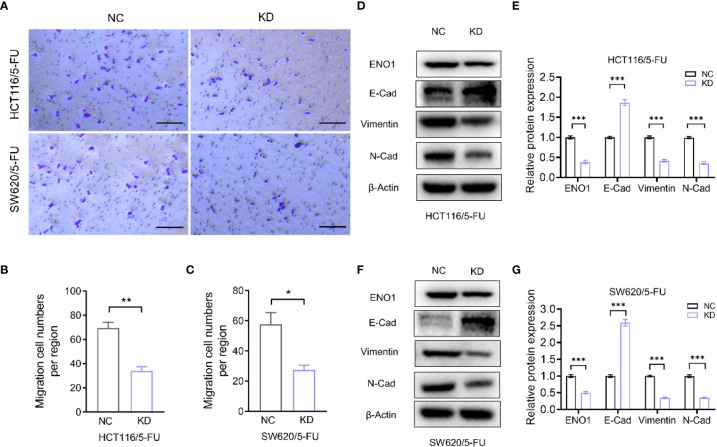
ENO1 knockdown inhibited the migration of resistant cells and was associated with the EMT process. **(A–C)** Display of representative photographs **(A)** in the ENO1-KD and NC groups and the number of migration cells in HCT116/5-FU cells (scale bar = 100 μm) **(B)** and SW620/5-FU cells **(C)**. **(D, F)** Western blotting of E-Cadherin, N-Cadherin, and Vimentin expression levels following knockdown of ENO1 in HCT116/5-FU cells **(D)** and SW620/5-FU cells **(F)**. **(E, G)** The bands were quantified and are presented as mean ± SEM of three independent experiments in HCT116/5-FU cells **(E)** and SW620/5-FU cells **(G)**. Statistical analyses were performed *via* two-tailed Student t test. *, P ≤ 0.05; **, P ≤ 0.01; ***, P ≤ 0.001.

**Figure 6:**
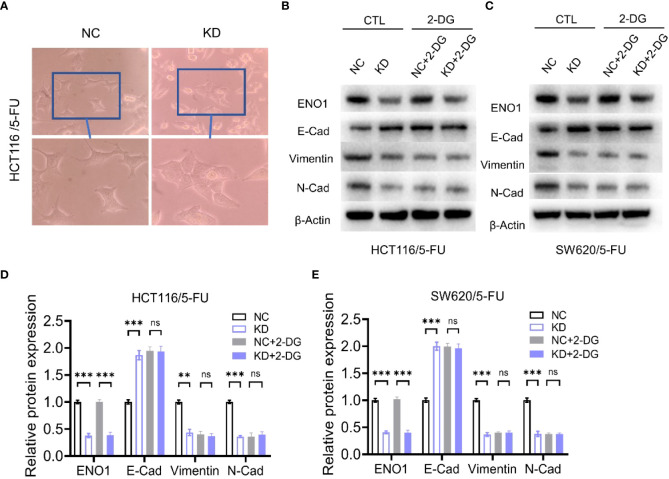
The regulation of the EMT process by ENO1 is associated with energy metabolism. **(A)** The morphological changes during the EMT process were suppressed in the ENO1-KD group compared with those in the NC group (observed by light microscope at 200× magnification). **(B, C)** Western blotting of E-Cadherin, N-Cadherin, and Vimentin expression in NC and ENO1-KD groups treated with or without 20 mM 2-DG for 4 h in HCT116/5-FU cells **(B)** and SW620/5-FU cells **(C)**. **(D-E)** The bands were quantified and are presented as mean ± SEM of three independent experiments in HCT116/5-FU cells **(D)** and SW620/5-FU cells **(E)**. Statistical analyses were performed *via* two-tailed Student t test. **, P ≤ 0.01; ***, P ≤ 0.001. ns, no significance.

Transwell assay displayed that ENO1 knockdown significantly inhibited the migration ability of HCT-116/5-FU and SW620/5-FU cells (**Figure 5A**). Migration cell numbers per region in the ENO1-KD group and NC group showed the same change (**Figures 5B, C**). To explore the potential mechanism underlying ENO1-mediated regulation of drug resistance in CRC cells, we intended to identify a correlation between ENO1 and cell characteristics such as angiogenesis, apoptosis, and cell cycle in lung, kidney, central nervous system, and other tumor tissues. On considering studies of tumor drug resistance and related pathways (20, 26), EMT may have a potential role in the regulation of ENO1 in drug-resistant CRC cells. Thus, the expressions of EMT pathway-related proteins in ENO1-KD and NC groups were detected through Western blotting. We found that the content of E-cadherin was increased, whereas the expression of Vimentin protein and N-cadherin protein in HCT116/5-FU cells was decreased in the ENO1-KD group compared with that in the NC group (**Figures 5D, E**). A similar change was observed in SW620/5-FU cells (**Figures 5F, G**). During the EMT process, the morphology of cells changes, making them more elongated and multipolar to better adapt to environmental changes. It was found that the morphological changes in the ENO1-KD group were suppressed compared with those in the NC group (**Figure 6A**). Furthermore, the CRC cells in the NC and ENO1-KD groups were treated with 20 mM 2-DG for 4 h, and it was found that the expression of E-cadherin was increased and the expressions of N-cadherin and Vimentin were decreased in the ENO1-KD group. However, no significant differences were observed in the expression of EMT-related proteins between the two groups after the treatment with 2-DG (**Figures 6B–E**). This revealed that 2-DG inhibited the effect of ENO1 on EMT progression, suggesting that the regulation of tumor proliferation, migration, and drug resistance by ENO1 may be related to energy metabolism.

### ENO1 knockdown inhibited CRC tumorigenesis *in vivo*

**Figure 7:**
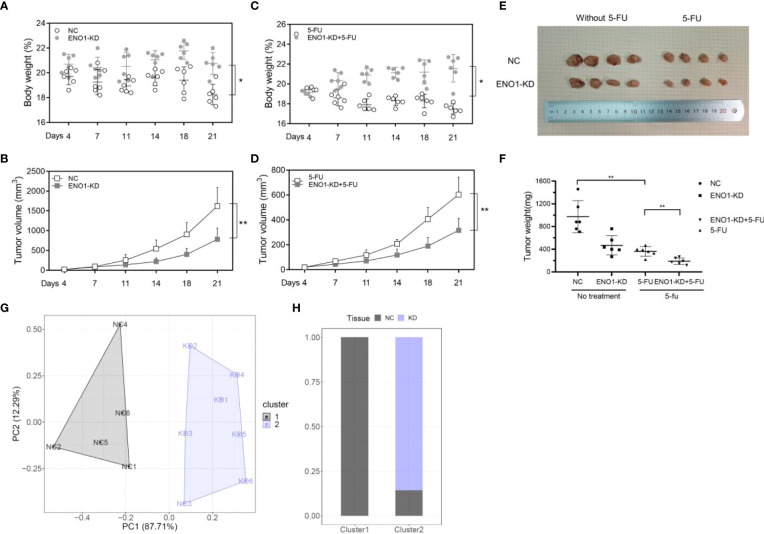
ENO1 knockdown suppresses tumor growth in colorectal cancer (CRC) *in vivo*. **(A, B)** Total body weight and tumor volume in ENO1-KD and NC groups. **(C, D)** Total body weight and tumor volume in ENO1-KD+5-FU and 5-FU groups. **(E)** Representative pictures of subcutaneous tumors harvested from the four groups. **(F)** The weights of tumor masses with or without treatment. **(G)** Stratification of mice into cluster 1 (grey) and cluster 2 (blue) according to tumor weight and ENO1 levels. **(H)** Percentage of NC and ENO1-KD mice in each cluster. Statistical analyses were performed *via* two-tailed Student t test. *, P ≤ 0.05; **, P ≤ 0.01.

To further verify the biological effect of ENO1 *in vivo*, we established a model by constructing subcutaneous tumors in mice using negative control and ENO1-knockdown HCT116 and SW620 cells. The mice were divided into four groups, and comparisons were made between them: ENO1-KD, NC, ENO1-KD with 5-FU, and NC with 5-FU. Depletion of ENO1 had a subtle effect on mouse body weight, but significantly inhibited the proliferative ability of CRC cells (**Figures 7A, B**). Similar phenomenon was observed after treatment with 5-FU. The tumor volume in ENO1-KD with 5-FU group was significantly decreased compared with that in the NC with 5-FU group (**Figures 7C, D**). Tumor weight in the ENO1-KD with 5-FU group was the lowest, followed by that in the NC with 5-FU group, and the weight in ENO1-KD group was lower than that in the NC group (**Figures 7E, F**). Next, cluster analysis was performed to evaluate the combined effects of tumor volume and tumor weight (**Figure 7G**). We found that all NC group mice were in Cluster 1, whereas 83.33% of ENO1-KD and 16.67% of NC group mice were in Cluster 2 (**Figure 7H**). This suggested that there is a significant difference between ENO1-KD and NC CRC cells *in vivo*.

## Discussion

Under the present circumstances, the incidence of CRC is increasing each year. Although comprehensive treatment has improved the overall survival of patients, patients with advanced CRC still face many challenges (12). Therefore, there is an urgent need to explore the mechanisms of proliferation, invasion, and metastasis in CRC and to find effective targets for inhibiting tumor progression.

ENO1 has been found to promote tumorigenesis, proliferation, and migration in many types of cancers. ENO1 regulates pancreatic cancer adhesion, invasion, and metastasis by controlling the expression of α-V/β-3 integrin (7). A significant increase has been observed in the roughness of cell membranes in ENO1-silenced PDA cells, along with a decrease in the levels of proteins involved in cell adhesion, including SαV/β3 integrin. These changes cause a decrease in cell migration and invasion *in vitro* and *in vivo*. ENO1 promotes lung cancer metastasis *via* HGFR and WNT signaling (27). A chimeric anti-ENO1 monoclonal antibody (ChenO1-22) can attenuate cancer cell invasion by inhibiting ENO1-mediated GSK3β inactivation to promote SLUG protein ubiquitination and block metastasis (27). Similarly, studies have shown that ENO1 monoclonal antibody plays a certain role in inhibiting the proliferation and invasion of cervical cancer cells (28). Other studies have shown that POMHEX, a small enolase inhibitor, selectively kills ENO1-deficient glioma cells at low nanomolar concentrations, suggesting that ENO1 acts as a promising target for inhibiting tumor progression (29). A similar trend was observed in bladder cancer, CRC, liver cancer, multiple myeloma, and many other diseases (30–32).

Although technological advances in radiotherapy and chemotherapy have improved local control and patient survival, treatment resistance, including chemotherapy resistance and radiation resistance, remain to be a major challenge in cancer research and treatment (33). There have been some studies on 5-FU-induced drug resistance in CRC. Some studies have found that 5-FU can promote the recurrence of CRC through the WNT pathway (34). Some specific drugs and small molecules have been known to modulate 5-FU resistance, but the specific molecular biological mechanism underlying this remains unclear (35, 36). Further, there are few studies on ENO1 affecting drug resistance in tumor cells. Overexpression of ENO1 has been shown to enhance tamoxifen resistance in breast cancer, and promote cisplatin resistance in gastric cancer cells by stimulating glycolysis, as well as methotrexate resistance in human HT29 cells (37). We knocked down ENO1 in 5-FU resistant HCT116 and SW620 cells, and found that the proliferation and migration ability of drug-resistant cells were reduced, which is an exploration of the genes related to 5-FU resistance.

Because ENO1 is a key enzyme in glucose metabolism, we attempted to explore whether ENO1-regulated tumor cell growth and drug resistance is associated with glucose metabolism. 2-DG is a glucose mimic that inhibits glycolysis and induces cell death through the formation and intracellular accumulation of 2-deoxyd-glucose-6-phosphate (2-DG6P) (38). Because of the ability of 2-DG to inhibit glycolysis and ATP synthesis, disrupt N-glycosylation of proteins, and decrease energy metabolism, it has been used as a radiation therapy option and adjuvant agent for chemotherapeutic drugs of various tumors (39, 40). The results of our experiment revealed that 2-DG reversed the effect of ENO1 on EMT progression, suggesting that ENO1-mediated regulation of tumor proliferation, migration, and drug resistance is associated with energy metabolism.

During EMT, cells lose epithelial cell-cell adhesion junctions and apical-basal cell polarity and acquire spindle cell morphology and migration ability (41). EMT is associated with resistance to various anticancer drugs. Previous studies have shown that targeting FGFR can successfully overcome EMT-mediated resistance in EGFR-mutant NSCLC (42, 43). It has been found that the expressions of EMT and stroma-related genes in urothelial carcinoma are associated with resistance to PD-1 blockade (44). EMT could induce gemcitabine resistance in pancreatic cancer and was found to be related to the characteristics of pancreatic cancer stem cells (20, 41, 45). However, the specific mechanism by which EMT mediates drug resistance was unclear. Our 2-DG comparative study showed that ENO1 could influence EMT process through the energy metabolism pathway.

In addition, we found that the application of new statistical and research methods to the existing results could identify signaling pathways more clearly, predict patient outcomes, and explore therapeutic targets with greater accuracy (46, 47). Compared with the traditional gene-centered methods, this method based on the concept of network markers can better detect the signal nodes and axes interconnecting many differentially expressed genes, and thus provide higher prediction accuracy (47). Single-cell multi-omics analysis has great potential for the mining of biological processes. This analysis method could measure the data pattern and the spatial cell environment at the same time, and will play a role in the realization of various biological goals, including the classification of cell types, construction of network nodes, and evaluation of biological processes (48).

In summary, our study confirmed that ENO1 expression was high in CRC and was associated with poor patient prognosis. By constructing 5-FU-resistant cells and knocking down ENO1 expression, we explored the effect of ENO1 alteration on drug resistance in CRC cells *in vivo* and *in vitro*. We found that the change was associated with the EMT process and regulation of energy metabolism. Thus, based on our findings, other articles on ENO1, and studies in other related fields, we believe that ENO1 may have application value in alleviating drug resistance in CRC.

## Data availability statement

The original contributions presented in the study are included in the article/supplementary material. Further inquiries can be directed to the corresponding authors.

## Ethics statement

The animal study was reviewed and approved by the First Affiliated Hospital of Suzhou University Ethics Committee. Written informed consent was obtained from the owners for the participation of their animals in this study.

## Author contributions

Conceptualization: XZ and BW. Methodology: XZ and BW. Investigation: JG, KZ, LW, HN, YZ, XW, YY and LJ. Analysis: JG, KZ and LW. Funding acquisition: XZ, BW and YY. Project administration: XZ and BW. Supervision: XZ and BW. Writing-original draft: JG, KZ and LW. Writing-review and editing: XZ and BW. All authors contributed to the article and approved the submitted version.
